# Cesarean Scar Pregnancy: An Experience of Three Cases with Review of Literature

**DOI:** 10.7759/cureus.2133

**Published:** 2018-02-01

**Authors:** Gulnaz Shafqat, Kumail Khandwala, Hina Iqbal, Shaista Afzal

**Affiliations:** 1 Department of Radiology, The Aga Khan University, Karachi.; 2 Department of Radiology, The Aga Khan University Hospital, Karachi.

**Keywords:** scar pregnancy, caesarean section, mri, ultrasound, ectopic pregnancy

## Abstract

Cesarean scar pregnancy (CSP), often considered the rarest form of ectopic pregnancy, is a result of implantation of the gestational sac into the fibrous tissue scar of a previous cesarean section. With an increase in the rate of cesarean sections, along with better awareness and improvement in sonographic diagnosis, the number and detection of scar pregnancies are on the rise. Because of its early invasion of the myometrium, usually in the first trimester, CSP is considered to be potentially lethal, leading to high risks of uterine rupture. We report a series of three cases of scar pregnancy that presented at different gestational ages and were managed by different methods. The aim of this case series is to share our experience with CSP, review previous literature, and emphasize on the radiological criteria to making a confident diagnosis. Diagnosis and management of CSP needs considerable expertise and a multidisciplinary approach to prevent complications.

## Introduction

Cesarean scar pregnancy (CSP), often considered the rarest form of ectopic pregnancy, is a result of implantation of the gestational sac into the fibrous tissue scar of a previous cesarean section (CS). Only 19 cases had been globally reported up to 2002 [1]. The incidence has been previously reported to be ranging from 1 in 1800 to 1 in 2200 pregnancies [2-3]. However, with an increase in cesarean sections, along with better awareness and improvement in sonographic diagnosis, the number and detection of scar pregnancies are on the rise. It is now predicted that approximately 6% of ectopic pregnancies in females with a history of at least one previous CS will be CSP [2].

The etiology and pathophysiology of CSP are not yet fully understood. Attributable factors have been thought to include endometrial and myometrial disruption, or formation of a microscopic tract due to trauma from a previous CS, resulting in scar implantation by the invading blastocyst [4]. Additionally, the larger the anterior uterine wall defect, the greater the odds of scar implantation, likely due to a greater surface area. CSP is considered to be even more aggressive than morbidly adherent placenta because of its early invasion of the myometrium, usually in the first trimester, leading to potentially high risks of uterine rupture [1-2]. One study suggests that CSPs share a similar histology and pathogenesis with early placenta accreta whereby implantation occurs where there is paucity of intervening decidual layer so that trophoblastic tissue invades the surrounding scar and myometrium [5]. CSP can lead to devastating complications, and therefore early diagnosis and prompt treatment are very important to reduce morbidity and mortality, and prevent potential hysterectomy.

We report a series of three cases of scar pregnancy that presented at different gestational ages and were managed by different methods. The aim of this case series is to share our experience with CSP, review previous literature, and emphasize on the radiological criteria to making a confident diagnosis.

## Case presentation

Case 1

**Figure 1:**
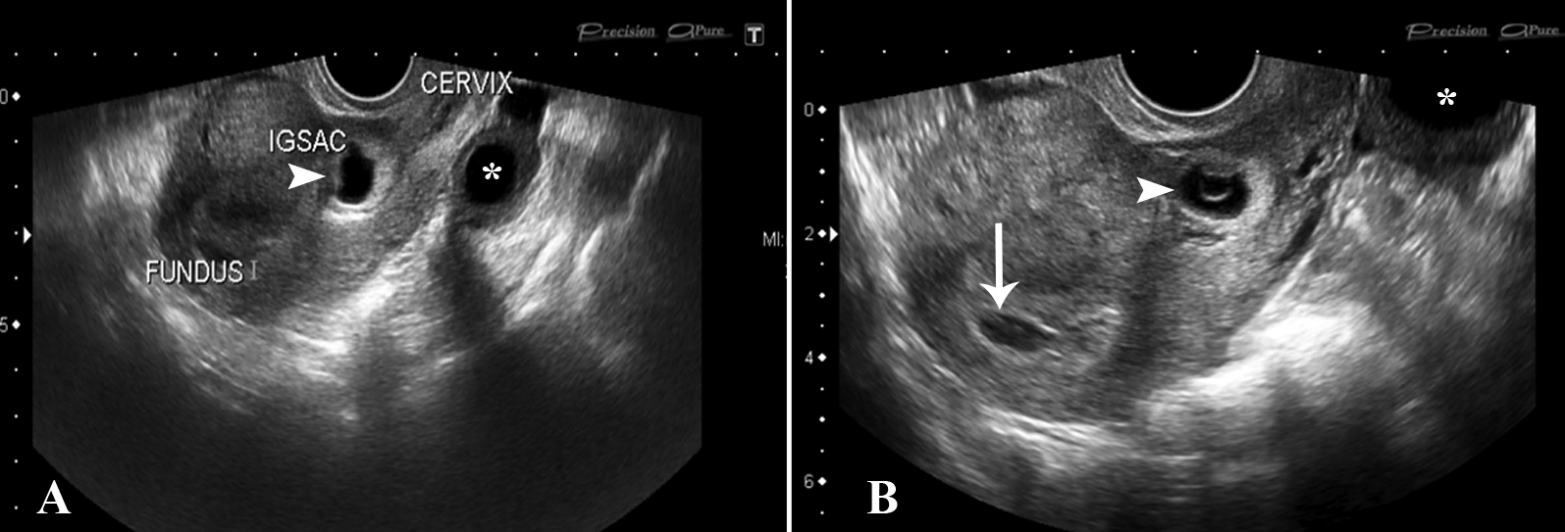
Initial TVS images showed the gestational sac to be located anteriorly in the lower uterine segment (arrowheads). The endometrial cavity was empty (arrow) and cervical os was closed. Corpus luteal cyst was seen in the left ovary (asterisk). TVS: Transvaginal scan

A 33-year-old female, Gravida (G)6 Para (P)4+2, with gestational amenorrhea of eight weeks, was admitted with a history of per vaginal spotting. She had a history of three previous cesarean sections. On initial transvaginal scan (TVS), a single intrauterine gestational sac was seen eccentrically near the cesarean section scar site in the lower anterior uterine segment. There was a minimal amount of fluid seen in the endometrial cavity which was otherwise empty. Cervix appeared normal and closed. The fetal pole was identified in the sac with crown-rump length (CRL) of 6.4 mm, corresponding to 6 weeks 4 days. Cardiac flicker was noted and normal yolk sac was seen. Corpus luteal cyst was noted in the left ovary (Figure 1).

**Figure 2:**
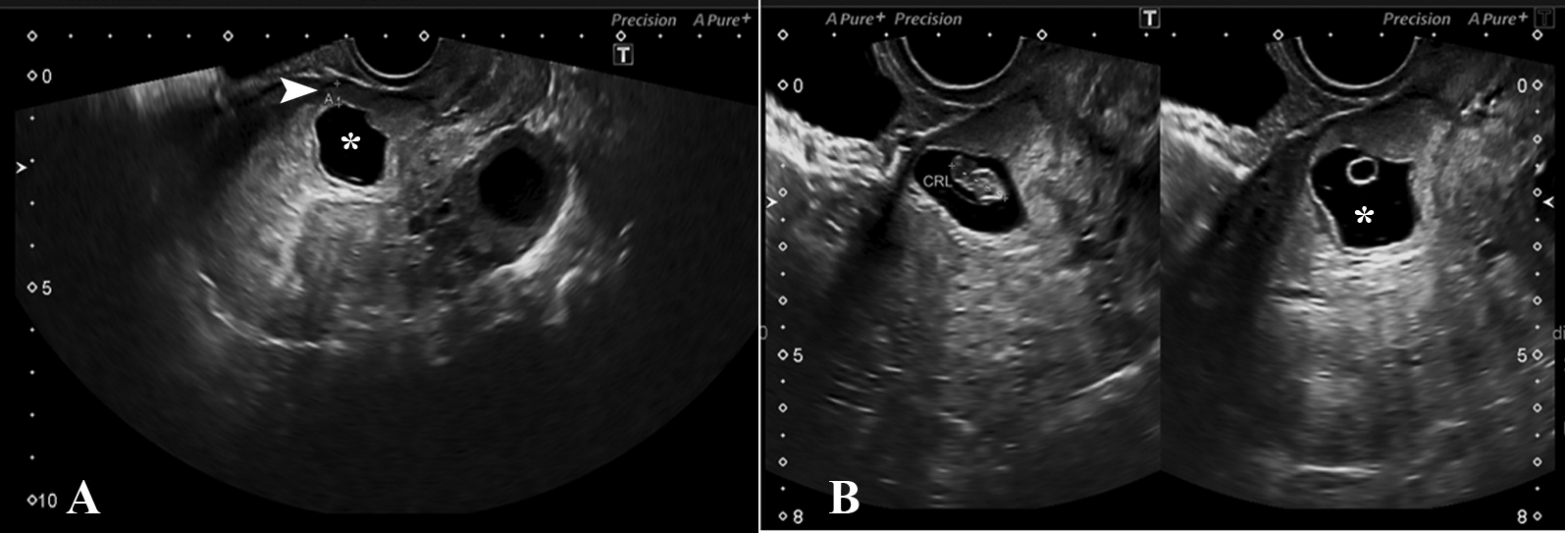
Eccentric gestational sac located anteriorly in the lower uterine segment (asterisk). Thin rim of myometrium was noted anteriorly measuring 4.5 mm (arrowhead). Live fetal pole and yolk sac visualized in the gestational sac.

High suspicion of CSP was raised, but the patient came back for a review ultrasound one week later for confirmation. This time the gestational sac at the scar site was growing towards the endometrial canal. Anterior myometrium between the gestational sac and the posterior wall of the urinary bladder was measuring 4.5 mm and was measuring the same on the initial TVS as well. The sac was growing towards the uterine cavity, and therefore this was representing type 1 or endogenic CSP (Figure 2). Exploratory laparotomy and resection of the lower uterine segment (approximately 1.5-2 cm) was done.

Case 2

**Figure 3:**
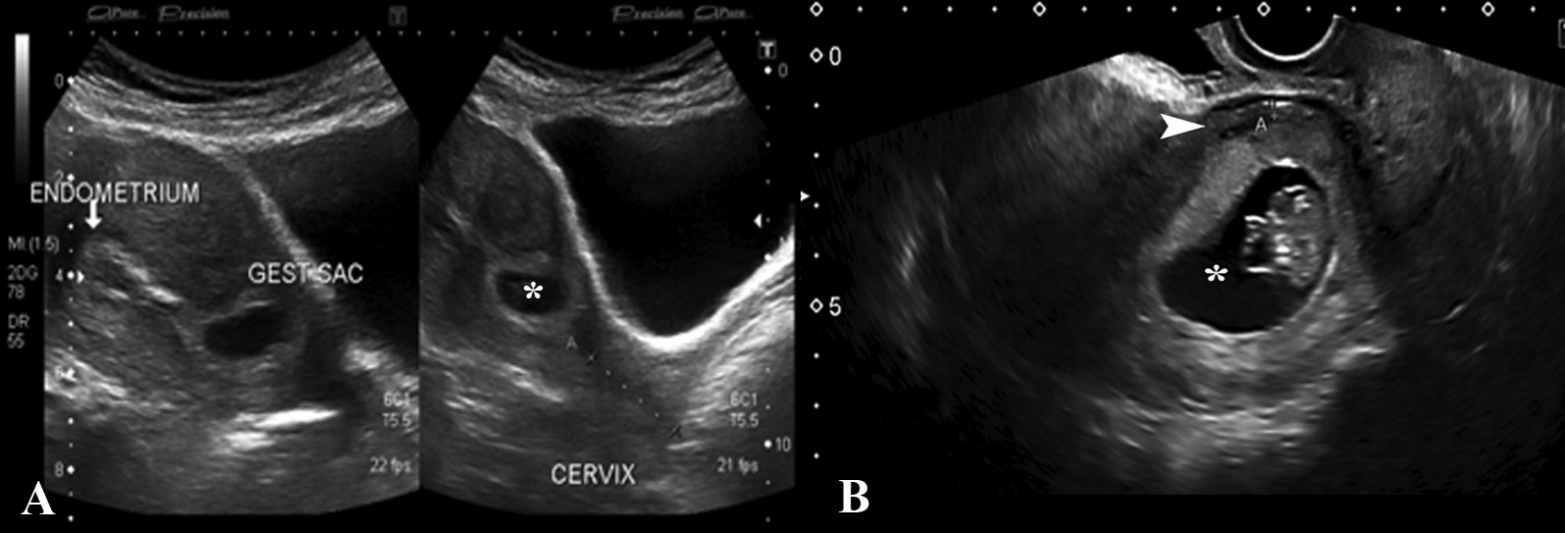
(A) Transabdominal scan images showing empty endometrial cavity and eccentrically placed gestational sac in the lower anterior uterine segment (asterisk). (B) TVS confirmed thin rim of myometrium anteriorly measuring 2.4 mm (arrowhead). TVS: Transvaginal scan

A 32-year-old female, G4 P3+0 with ten weeks gestational amenorrhea and history of three previous cesarean sections, was admitted to the emergency room (ER) with complaints of left lumbar pain for two days. Transabdominal and transvaginal scan showed single intrauterine gestational sac in the lower half of the uterine body close to the scar site. A fetal pole was identified with CRL of 3.58 mm, corresponding to 10 weeks and 3 days. Cardiac flicker was noted and normal yolk sac was seen. The cervical internal os was closed. Only a thin rim of myometrium measuring 2.4 mm was identified anterior to the gestational sac. The endometrial cavity in the upper-uterine segment was empty (Figure 3).

**Figure 4:**
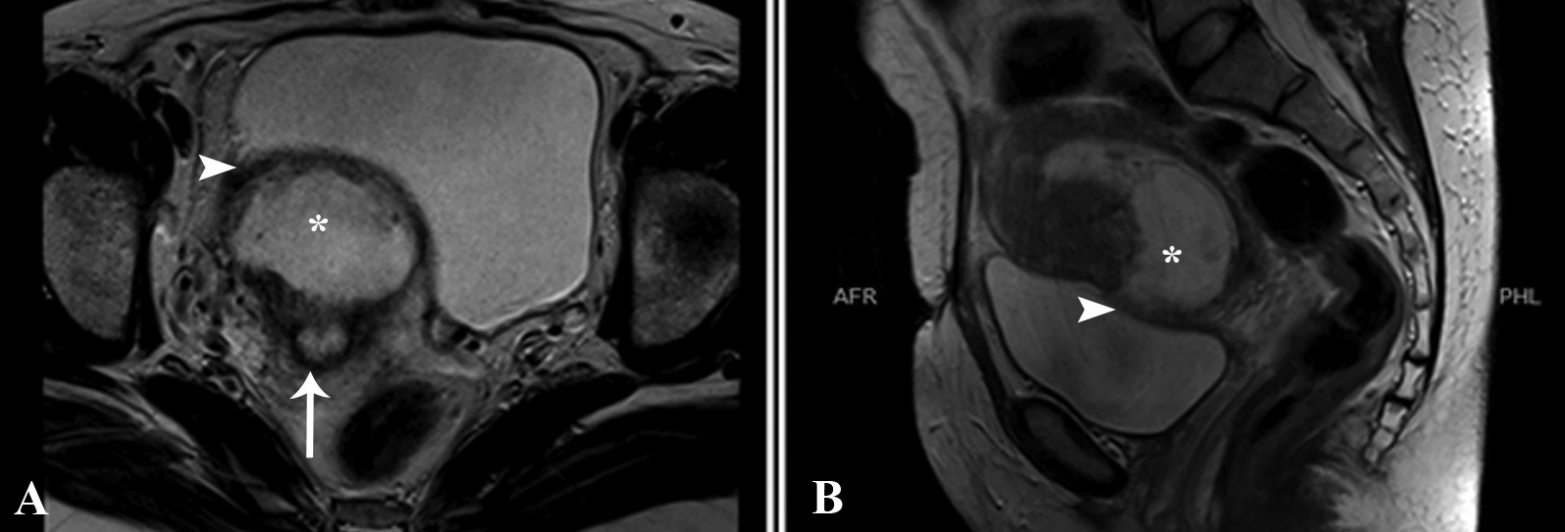
Axial and sagittal T2-weighted MRI from the pelvis showing gestational sac with fetal pole bulging anteriorly into the urinary bladder at the previous scar (asterisk). No myometrium identified adjacent to the sac where it was covered by thin hypointense layer of serosa (arrowhead). Endometrium is visualized separately (arrow). MRI: Magnetic resonance imaging

The patient subsequently underwent magnetic resonance imaging (MRI) examination for confirmation. MRI showed an intrauterine gestational sac in the lower uterine segment bulging through the anterior uterine wall at the site of the cesarean scar. No myometrium was identified adjacent to the sac where it was covered by a thin hypointense layer of serosa. There was no invasion of the urinary bladder. The cervical stroma and endocervix returned normal signals. Since the gestational sac was growing towards the bladder, this was representing type 2, or exogenic CSP (Figure 4).

The patient was initially managed medically with injection methotrexate (MTX); however, due to non-progress and hemodynamic instability, surgery was planned. Preoperatively, bilateral uterine arteries were embolized. Laparotomy was done. Intraoperative findings included adhesions between the anterior abdominal wall and omentum. The lower anterior uterine segment was ballooned and thinned out at previous scar site. The urinary bladder was closely attached to the uterine surface. Due to excessive bleeding and fragile tissue, total abdominal hysterectomy was carried out.

Case 3

**Figure 5:**
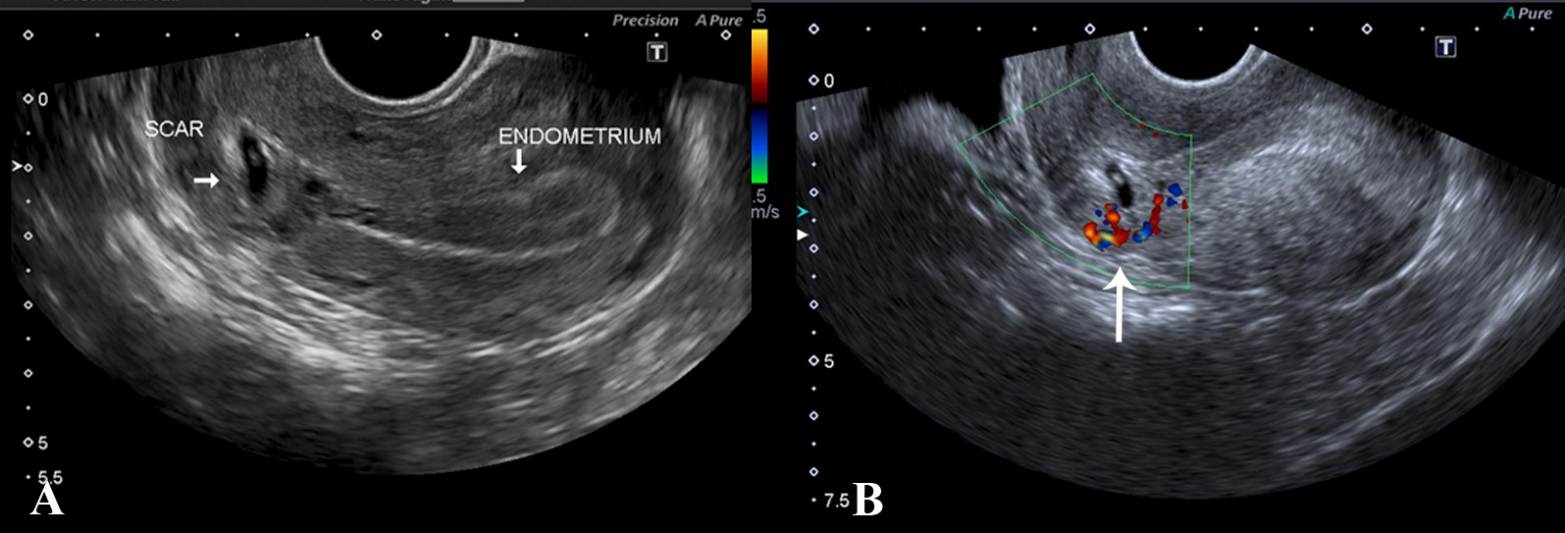
(A) TVS showing retroverted uterus with empty endometrial cavity and low-lying gestational sac containing fetal pole, close to the scar site. (B) Increased vascularity was noted around the gestational sac on Doppler (arrow). TVS: Transvaginal scan

A 28-year-old female, G6 P3+2 with gestational amenorrhea of six weeks and history of three previous cesarean sections presented with pervaginal spotting. Transvaginal scans showed retroverted uterus with empty uterine body and fundus as well as development of a gestational sac in the anterior part of the lower uterine segment. The sac was slightly eccentrically placed, lying just above the internal os in close proximity to the surgical scar. There was significant myometrial thinning anterior to it, with thickness measuring 5 mm. A fetal pole was identified measuring 0.20 cm corresponding to five weeks and five days. Cardiac flicker was noted and normal yolk sac was seen. Additionally, there was increased vascularity around the sac on color Doppler (Figure 5).

This gestational sac was appearing to grow towards the cervico-isthmic cavity, and it was likely representing type 1 or endogenic CSP. Injection MTX was given for medical termination of pregnancy with serial beta human chorionic gonadotropin (hCG) monitoring. She subsequently underwent hysteroscopy and uterine evacuation. Post-operative period was unremarkable and post-procedural ultrasound showed no evidence of retained products of conception (RPOC). She was discharged in a stable state.

## Discussion

Patients with CSP may be asymptomatic or present with mild vaginal bleeding and/or abdominal discomfort. The clinical diagnosis of an early CSP can be very difficult and may occasionally be delayed until uterine rupture occurs and leads to potentially fatal hemorrhage. Moreover, a cesarean scar pregnancy can also be easily misdiagnosed as a low intrauterine pregnancy, cervical ectopic pregnancy, or an inevitable miscarriage. There is no correlation with the number of previous CS and the risk of developing a CSP, since it can occur after only one CS [4]. However in our case series, there was history of at least three prior cesarean sections in all three patients.

Ultrasound imaging has remained the principal modality of choice for diagnosing CSP. A combined transabdominal and transvaginal ultrasound approach has provided a high accuracy rate in the diagnosis of CSP. A transabdominal pelvic ultrasound scan allows a panoramic view of the uterus and its relation to the bladder, and may be useful in later stages of pregnancy. However, transvaginal ultrasound has a reported sensitivity of 84.6% for detection of CSP in early pregnancy [6]. We were able to accurately diagnose CSP as early as five weeks and five days on TVS.

CSPs tend to be located eccentrically in the anterior lower uterus just superior to the cervix. Described sonographic features of CSP include an empty uterine cavity, empty and closed cervical canal, gestational sac (triangular, round or oval shaped) within the anterior portion of the lower uterine segment, a diminished or absent myometrial layer between the gestational sac and the bladder, and a discontinuity in the anterior wall of the uterus being demonstrated on a sagittal view of the uterus when the direction of the ultrasound beam runs through the amniotic sac [1-2, 7]. These criteria help in differentiating CSP from cervicoisthmic implantation, cervical pregnancy and spontaneous abortion in progress.

In addition, a prominent peritrophoblastic flow is demonstrated around the gestational sac on color Doppler. High velocity (peak velocity >20 cm/s) and low impedance blood flow (pulsatility index <1) suggests functional trophoblastic/placental circulation, and this also helps to distinguish CSP from a non-viable detached intrauterine pregnancy [2-3]. Other reports have shown resistance index (RI) <0.5 and a peak value ratio of systolicto-diastolic (S/D) blood flow as being <3 [8]. Some authors have suggested that the “sliding sign” (i.e., occurs when pressure is applied to the cervix using the probe, causing the gestational sac to slide against the endocervical canal in cases of miscarriage) is absent in cases of cervical ectopic pregnancies and CSPs [9].

MRI is also a valuable imaging tool for the diagnosis of CSP. MRI provides vital information in cases in which ultrasound is indeterminate, if the uterine anatomy is distorted due to the presence of large fibroids, or if the pregnancy has progressed to a later stage [10]. In addition, multiplanar capability of MRI can clearly delineate adjacent organ involvement and therefore be helpful in orienting the surgeon should operative management be warranted.

Based on imaging findings and pregnancy progression, CSP can be classified into two types: Type 1, or endogenic CSP, is when implantation occurs on the scar site and the gestational sac grows towards the cervico-isthmic or uterine cavity; Type 2, or exogenic CSP, occurs when the gestational sac is deeply embedded in the scar and the surrounding myometrium and grows towards the urinary bladder. In exogenic types, a layer of myometrium may be seen between the gestational sac and the bladder at an earlier stage; this becomes thin and eventually disappears, with bulging of the gestational sac through the gap as the pregnancy progresses. Thus this type of CSP carries a greater risk of earlier uterine rupture. In two-thirds of cases, the thickness of the scar may be less than 5 mm [9]. Case two in our series was an exogenic CSP, which resulted in the patient's hemodynamic instability and eventually required vascular intervention along with operative management.

There is no agreement on the preferred management protocol to date. Treatment options are individualized according to gestational age and presentation, usually with the aim of removing the gestational sac, preventing hemorrhage and retaining future fertility. Medical methods entail systemic or local administration of MTX, injection of potassium chloride into the sac, or injection of mifepristone with monitoring of beta hCG levels. Surgical methods include uterine suction curettage, hysteroscopic evacuation or local resection of the mass, and laparoscopic, transvaginal or open hysterectomy, with the latter indicated for patients with hemodynamic instability. Interventional radiology techniques like uterine artery embolization (UAE) have also been successfully employed. UAE can also be used preoperatively to decrease the risk of hemorrhage. This combined approach may be a feasible option for those patients who present with hemorrhage but maintain a desire to preserve future fertility [6].

## Conclusions

In summary, we report three cases of cesarean scar pregnancy that were managed with individualized treatment methods based on the clinical presentation, gestational age, and type of pregnancy on imaging. Ultrasound has remained the principal modality of choice for diagnosing CSP; however, MRI may guide the surgeon should operative management be warranted. Diagnosis and management of CSP can sometimes be challenging and requires a multidisciplinary approach to prevent associated complications. Prompt and accurate diagnosis of CSP and follow-up are required to reduce overall maternal morbidity and mortality.
